# Effectiveness of Distraction Techniques in Managing Pediatric Dental Patients

**DOI:** 10.5005/jp-journals-10005-1582

**Published:** 2019

**Authors:** Madhuri Khandelwal, Raghavendra M Shetty, Sujata Rath

**Affiliations:** 1 Department of Pedodontics and Preventive Dentistry, Chhattisgarh Dental College and Research Institute, Rajnandgaon, Chhattisgarh, India; 2 Department of Preventive and Pediatric Dentistry, Gulf Medical University, Ajman, UAE; 3 Department of Pedodontics and Preventive Dentistry, Hi-Tech Dental College, Bhubaneswar, Odisha, India

**Keywords:** Behavior management, Dental anxiety, Distraction

## Abstract

**Aim:**

Children having dental anxiety usually hesitate to seek dental care which can result in poor oral health and may lead to expensive and complex dental treatment in the future. The aim of the present study is to compare and evaluate the effectiveness of various distraction techniques in managing pediatric dental patients.

**Materials and methods:**

Eighty healthy children selected for the study were randomly divided into 4 groups with 20 children in each group. Group I was termed as the control group; in group II, the audio distraction technique was used. Group III received audio–video distraction (AVD) by means of a chair-mounted audio–video device and group IV received AVD by means of a ceiling-mounted television. Each child had four dental visits. Child's anxiety in each visit was assessed using four parameters: RMS pictorial scale (RMS-PS), Venham picture test (VPT), pulse rate, and oxygen saturation.

**Results:**

Ceiling-mounted AVD was found to be the most effective in reducing the anxiety followed by chair-mounted AVD. Audio distraction was found to be the least effective but was better than the control group.

**Conclusion:**

The AVD technique is simple, passive, and noninvasive means of behavior management and can be used alternatively in managing anxious pediatric dental patients.

**How to cite this article:**

Khandelwal M, Shetty RM, *et al.* Effectiveness of Distraction Techniques in Managing Pediatric Dental Patients. Int J Clin Pediatr Dent 2019;12(1):18–24.

## INTRODUCTION

A young child's emotional and behavioral response to dental treatment is a matter of serious concern to pediatric dentists and researchers. The child's fearful or uncooperative behavior may impede the efficient delivery of dental care and compromise the quality of treatment provided. If not adequately resolved, a persistent negative response pattern may emerge which functions as a barrier to routine dental care.^1^

A range of fear management techniques have been described in the literature and American Academy of Pediatric Dentistry (AAPD) has described basic concepts as basic behavior guidance such as communication, tell show do, voice control, nonverbal communication, positive reinforcement, distraction and parental absence/presence, and advanced behavior guidance such as protective stabilization, sedation, and general anesthesia.^2^

Clinical and research reports provide varying degrees of support for the effectiveness of each method. However, some methods also involve significant disadvantages. Physical restraint and pharmacological intervention may involve a potential physical hazard to the child. Modeling and reinforcement are time consuming. In contrast, distraction methods can be safe, effective, and economical for the clinician to use.^1^ Hence, the present study was undertaken to evaluate and compare the effectiveness of various distraction techniques in the management of anxious pediatric dental patients.

## MATERIALS AND METHOD

### Source of Data

A total of 80 healthy children among 1,040 children between age range 4 and 10 years, reporting to the department of pediatric dentistry for the first dental visit with their parents/guardian, were selected for the study. The research protocol of the study was reviewed and approved by the ethical committee of the institution. Informed consent was obtained from parents/guardians.

Children during their first visit with definite indications of oral prophylaxis, restorations, and local anesthesia (LA) administration either for the extraction or pulp therapy were included in the study. Children with previous dental experience and with any sort of mental or physical disability were excluded from the study.

### Methodology

The selected children for the study were randomly divided into four groups which were as follows:

Group I: Twenty children on whom treatment was performed under normal dental setup without using any distraction technique (control group).

**Figs 1A and B:**
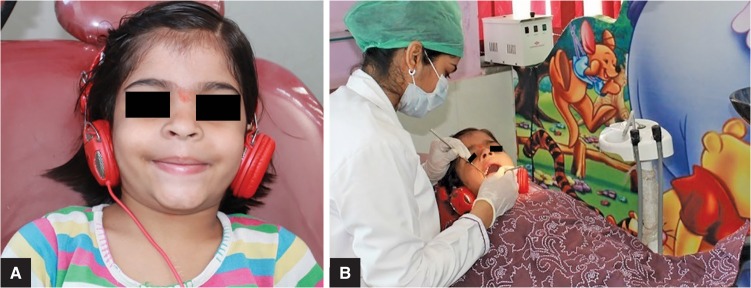
(A) Child with headphones in the audio group; (B) Child undergoing treatment in the audio group

**Figs 2A and B:**
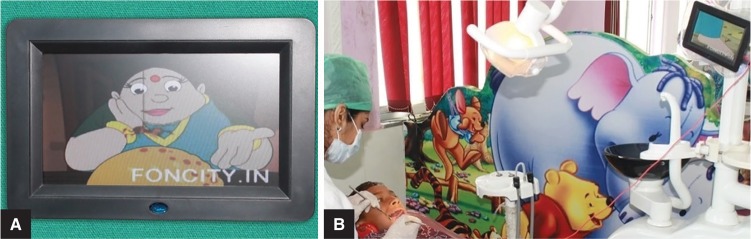
(A) Chair-mounted audio–video device; (B) Child undergoing treatment in the chair-mounted audio–video group

Group II: Twenty children on whom treatment was performed using the audio distraction technique through headphones. Children listened to selected popular songs (audio group, Fig. 1).

Group III: Twenty children on whom treatment was performed under AVD through a chair-mounted audio–video device with headphones. Selected popular cartoon films were played in the device (chair-mounted audio–video group, Fig. 2).

**Figs 3A and B:**
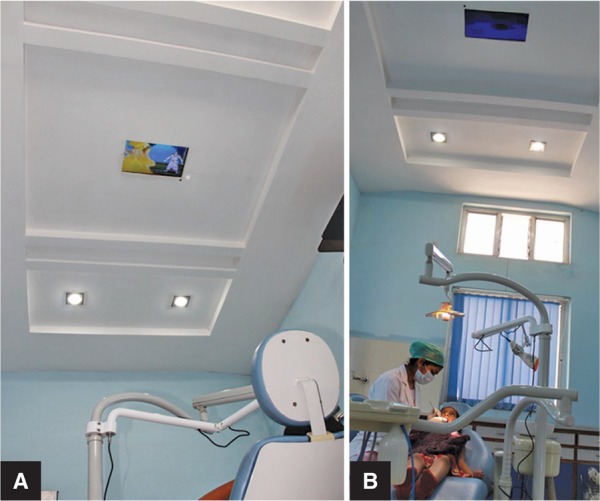
(A) Ceiling-mounted television; (B) Child undergoing treatment in the ceiling-mounted audio–video group

Group IV: Twenty children on whom treatment was performed under AVD through a ceiling-mounted television. Selected popular cartoon films were played on the television (ceiling-mounted audio–video group, Fig. 3).

Every child from each group had a total of four dental visits:

- The first visit included screening and intraoral examination.
- The second visit included oral prophylaxis.
- The third visit included cavity preparation followed by restoration.
- The fourth visit included administration of LA followed by extraction or pulp therapy.

### Assessment of Dental Anxiety

Child's anxiety level in each visit was assessed using various methods which were as follows:

- RMS pictorial scale (RMS-PS)^3^
- Venham picture test (VPT)^4^
- Pulse rate
- Oxygen saturation

### Statistical Analysis

Statistical analyses were performed using the SPSS version 17 software. Analysis of variance (ANOVA) followed by the Tukey's *post hoc* test was applied to compare the four groups during the four visits.

## RESULTS

### RMS-PS

There was a gradual decrease in the mean RMS-PS scores from the first visit (examination) to the third visit (cavity preparation) in group I (control group). The RMS-PS score increased in the fourth visit (LA administration) indicating that there was an increase in the anxiety (Table 1).

The mean RMS-PS score during examination, prophylaxis, and cavity preparation across the groups showed no statistically significant difference. But during the fourth visit (LA administration), a statistically significant difference was seen when compared across the groups.

**Table 1:** Inter- and intragroup comparison of RMS-PS

*Visit*	*Group I (mean ± SD)*	*Group II (mean ± SD)*	*Group III (mean ± SD)*	*Group IV (mean ± SD)*	*Comparison between groups*
Examination (first visit)	2.05 ± 0.85	2.05 ± 1.35	2.05 ± 1.05	1.80 ± 0.52	*F* = 0.31
					*p* = 0.81
Prophylaxis (second visit)	1.85 ± 0.76	1.85 ± 0.75	1.70 ± 1.00	1.55 ± 0.48	*F* = 0.69
					*p* = 0.55
Cavity preparation (third visit)	1.80 ± 0.85	1.80 ± 0.88	1.60 ± 0.81	1.50 ± 0.60	*F* = 0.71
					*p* = 0.54
LA administration (fourth visit)	2.25 ± 1.01	2.15 ± 1.03	1.50 ± 0.88	1.40 ± 0.75	*F* = 4.46
					*p* = 0.006*
Comparison in between visits	*F* = 1.11	*F* = 0.51	*F* = 1.29	*F* = 1.62	
	*p* = 0.35	*p* = 0.67	*p* = 0.28	*p* = 0.13	

* Significant. SD, standard deviation

**Table 2:** Intragroup comparison of RMS-PS during LA administration

*Groups*			*F value*	*p value*
Group I	vs	Group II	0.09	0.75
		Group III	6.26	0.017*
		Group IV	9.13	0.004*
Group II	vs	Group III	4.60	0.038*
		Group IV	6.93	0.012*
Group III	vs	Group IV	0.15	0.70

* Significant

On further comparison, when group I was compared with group III (chair-mounted audio–video group) and group IV (ceiling-mounted audio–video group), a statistically significant difference was seen which indicated that there was more reduction in anxiety scores. Similarly, a statistically significant difference was seen when group II was compared with group III and with group IV (Table 2). However, there was no statistical significance seen between groups III and IV.

### VPT

Similar to the RMS-PS score only during the fourth visit (LA administration), there was a statistically significant difference when compared across the groups (Table 3). On intergroup comparison of the fourth visit scores, it was seen that groups III and IV showed a statistically significant difference in anxiety scores when compared to group I. Similarly, a statistically significant difference was also seen when group II was compared with group III and with group IV scores. However, there was no statistical significance seen between groups III and IV (Table 4).

### Mean Pulse Rate

The pulse rate was recorded using a fingertip pulse oximeter (NiscoMed). The pulse rate was measured four times during the session and the mean pulse rate was calculated.

#### Group I (Control Group)

There was a significant increase in the mean pulse rate in the second, the third, and the fourth visit. A statistically significant difference was seen between the first and the second visit and between the first and the third visit. A highly significant difference was seen between the first and the fourth visit (*p* ≤ 0.001) (Table 5). The significant difference in the mean pulse rate indicated an increase in the anxiety in group I.

#### Group II (Audio Group)

There was a statistically significant difference seen between the first and the fourth visit which indicated an increase in anxiety in group II from the first visit to the fourth visit (Table 5).

#### Group III (Chair Mounted Audio–Video) and Group IV (Ceiling Mounted Audio–Video)

There was no statistically significant difference seen when the pulse rate was compared during the subsequent four visits (Table 5).

On intergroup comparison during the fourth visit, a statistically significant difference was seen when compared across the groups (Table 6). When group I was compared with groups III and IV, a statistically significant difference was seen between them which indicated that there was more reduction in anxiety when chair-mounted audio–video and ceiling-mounted audio–video distraction (AVD) techniques were used when compared with the control group.

**Table 3:** Inter- and intragroup comparison of VPT

*Visit*	*Group I (mean ± SD)*	*Group II (mean ± SD)*	*Group III (mean ± SD)*	*Group IV (mean ± SD)*	*Comparison between groups*
First visit	3.35 ± 2.48	3.35 ± 2.68	2.65 ± 1.92	1.70 ± 2.02	*F* = 2.31
					*p* = 0.08
Second visit	2.75 ± 2.06	2.70 ± 2.06	2.15 ± 2.08	1.60 ± 2.08	*F* = 1.36
					*p* = 0.26
Third visit	2.45 ± 2.32	2.25 ± 1.98	2.00 ± 1.89	1.30 ± 1.29	*F* = 1.38
					*p* = 0.25
Fourth visit	3.25 ± 2.55	2.65 ± 2.06	1.55 ± 1.35	1.00 ± 1.57	*F* = 5.56
					*p* = 0.003*
Comparison in- between visits	*F* = 0.64	*F* = 0.84	*F* = 1.22	*F* = 0.63	
	*p* = 0.58	*p* = 0.47	*p* = 0.30	*p* = 0.59	

* Significant

**Table 4:** Intragroup comparison of VPT during LA administration

*Groups*			*F value*	*p value*
Group I	vs	Group II	0.41	0.67
		Group III	6.94	0.012*
		Group IV	11.29	0.002*
Group II	vs	Group III	3.98	0.049*
		Group IV	8.11	0.007*
Group III	vs	Group IV	1.41	0.24

* Significant

A statistically significant difference was seen between groups II and III and in between groups II and IV indicating that chair-mounted audio–video and ceiling-mounted AVD techniques were better than the audio distraction technique in reducing anxiety.

When the mean pulse rate of groups III and IV was compared, the pulse rate was higher in group III. However, no statistically significant difference was seen between them (Table 6).

### Oxygen Saturation

On intragroup and intergroup comparison, no statistically significant difference was seen when compared in their subsequent visits within the group and across the groups (Table 7).

### Overall Inter- and Intragroup Comparison of All the Parameters

The difference in overall RMS-PS scores was highly significant across the groups (*F* = 6.45, *p* <0.001). The highest anxiety scores were seen in group I followed by groups II, III, and IV (Table 8). A highly significant difference was seen when scores of groups I and II were compared with group IV. A significant difference was seen between the scores of groups I and III and between groups II and III. No statistically significant difference was seen when RMS-PS scores of groups III and IV were compared (Table 9).

When the overall mean VPT scores of all four visits in all four groups were compared, a highly significant difference was seen. Group IV has shown the least anxiety score followed by groups III, II, and I indicating that the ceiling-mounted AVD was the most efficient in reducing anxiety followed by the chair-mounted AVD technique, whereas the audio distraction technique was found to be the least effective (Table 8). A highly significant difference was seen between the VPT scores of groups I and IV and between groups II and IV (*p* ≤ 0.001). A statistically significant difference was also seen between the VPT scores of groups I and III and groups II and III (Table 9).

**Table 5:** Inter and intragroup comparison of pulse rate

*Visit*	*Group I (mean ± SD)*	*Group II (mean ± SD)*	*Group III (mean ± SD)*	*Group IV (mean ± SD)*	*Comparison between groups*
First visit	100.45 ± 14.54	100.20 ± 20.49	100.30 ± 11.90	100.40 ± 15.93	*F* = 0.001
					*p* = 1.00
Second visit	111.15 ± 15.28	111.25 ± 16.90	106.15 ± 9.01	102.10 ± 15.54	*F* = 1.84
					*p* = 0.26
Third visit	112.81 ± 13.84	111.60 ± 16.72	104.15 ±10.55	103.25 ± 15.93	*F* = 2.34
					*p* = 0.08
Fourth visit	117.45 ± 15.76	116.55 ± 13.75	108.55 ± 11.26	105.20 ± 11.97	*F* = 4.09
					*p* = 0.010*
Comparison in- between visits	*F* = 4.67, *p* = 0.005*	*F* = 3.24, *p* = 0.026*	*F* = 2.11	*F* = 0.77	
	1st vs 2nd *p* = 0.02*	1st vs 4th *p* = 0.005*	*p* = 0.10, NS	*p* = 0.36, NS	
	1st vs 3rd *p* = 0.009*				
	1st vs 4th *p* ≤ 0.001*				

* Significant. NS, nonsignificant

**Table 6:** Intragroup comparison of pulse rate during LA administration

*Groups*			*F value*	*p value*
Group I	vs	Group II	0.03	0.48
		Group III	4.22	0.047*
		Group IV	7.66	0.009*
Group II	vs	Group III	4.05	0.05*
		Group IV	7.75	0.008*
Group III	vs	Group IV	0.83	0.36

* Significant

When the overall mean pulse rate during all four visits in all four groups was compared, a highly significant difference was seen. The mean pulse rate was the least in group IV and the highest in group I (Table 8). A highly significant difference was seen between the mean scores of groups I and III (*p* ≤ 0.001) and groups I and IV. A statistically significant difference was also seen between the mean scores between groups II and III and in between groups II and IV (Table 9).

## DISCUSSION

Dental anxiety has been the primary reason for not seeking dental care. Children who experience high levels of dental anxiety tend to have higher caries experience.^5^ These fears and anxieties should be addressed or it can affect patients’ oral health, and may result in costly dental treatments that could have been avoided through preventive care. So, there is a need for proper assessment of dental anxiety followed by a treatment session in a pleasant or less stressful dental setting to relieve fear and anxiety.

The age group of the patients selected in the present study belonged to 4–10 years as children show disruptive or negative behavior in this age group and are difficult to manage.^6,7^ RMS-PS is a newer anxiety assessment scale.^3^ The validity of the RMS-PS in the dental setting in the assessment of a child's dental anxiety was supported by its strong correlation with the VPT scores. The RMS-PS has many advantages over the former anxiety assessment measures. It offers a simple, quick, efficient evaluation of anxiety for a pediatric dental patient.

**Table 7:** Inter and intragroup comparison of oxygen saturation

*Visit*	*Control group, mean ± SD*	*Audio group, mean ± SD*	*Chair-mounted AV group, mean ± SD*	*Ceiling-mounted AV group, mean ± SD*	*Comparison between groups*
First visit	98.2 ± 0.74	98.2 ± 0.43	98.3 ± 0.38	98.5 ± 0.23	*F* = 1.7
					*p* = 0.17
Second visit	98.4 ± 0.30	98.2 ± 0.30	98.3 ± 0.40	98.3 ± 0.50	*F* = 0.90
					*p* = 0.44
Third visit	98.3 ± 0.48	98.4 ± 0.48	98.1 ± 0.37	98.4 ± 0.57	*F* = 1.73
					*p* = 0.16
Fourth visit	98.1 ± 0.56	98.3 ± 0.35	98.2 ± 0.47	98.3 ± 0.26	*F* = 1.01
					*p* = 0.39
Comparison in- between visits	*F* = 1.12	*F* = 1.16	*F* = 1.10	*F* = 1.37	
	*p* = 0.34	*p* = 0.32	*p* = 0.35	*p* = 0.25	

**Table 8:** Overall mean scores of various parameters across the groups

*Visit*	*Group I, mean ± SD*	*Group II, mean ± SD*	*Group III, mean ± SD*	*Group IV, mean ± SD*	*Comparison between groups*
RMS-PS	1.98 ± 0.87	1.96 ± 0.09	1.71 ± 0.91	1.57 ± 0.61	*F* = 6.45
					*p* ≤ 0.001**
VPT	2.95 ± 2.36	2.73 ± 2.28	2.08 ± 1.87	1.40 ± 1.51	*F* = 9.45
					*p* ≤ 0.001**
Pulse rate	111.46 ± 15.50	109.90 ± 16.96	104.75 ± 9.98	102.73 ± 14.80	*F* = 6.47
					*p* ≤ 0.001*
Oxygen saturation	98.25 ± 0.52	98.27 ± 0.39	98.22 ± 0.40	98.37 ± 0.39	*F* = 0.84
					*p* = 0.14

* Significant.

** Highly significant

**Table 9:** Overall intergroup comparison of RMS-PS, VPT and pulse rate across the groups

*Groups*			*RMS-PD*			*VPT*			*Pulse rate*	
			*F value*	*p value*		*F value*	*p value*		*F value*	*p value*
Group I	vs	Group II	0.04	0.83		0.36	**0.55**		0.36	0.54
		Group III	3.67	0.05*		6.67	0.011*		8.19	≤0.001**
		Group IV	11.91	≤0.001**		24.48	≤0.001**		13.27	≤0.001**
Group II	vs	Group III	11.91	≤0.001**		24.48	≤0.001**		13.27	≤0.001**
		Group IV	32	≤0.001**		18.92	≤0.001**		8.11	0.005*
Group III	vs	Group IV	1.30	0.25		6.40	**0.12**		0.03	0.86

* Significant.

** Highly significant

VPT^4^ is one of the most commonly used picture scale. It is a self-report measure that permits measurement of the state of anxiety of children when visiting a dentist. VPT is considered as one of the reliable measures of self-reported anxiety in children.^8–10^

A pulse oximeter is one of the most acceptable methods for measuring the physiological changes as it gives continuous percentage measurements of the patient's arterial hemoglobin oxygenation as well as the pulse rate.^11,12^ Hence, in the present study, RMS-PS, VPT, pulse rate, and oxygen saturation were used for the assessment of anxiety.

The observations from our study indicated that the RMS-PS gave a statistically significant result. The findings showed that the chair-mounted AVD technique and the ceiling-mounted AVD technique have shown a reduction in anxiety when compared with the audio distraction technique and when no distraction was used (control group).

VPT in the present study also gave similar results as RMS-PS. The findings showed that the chair-mounted AVD technique and the ceiling-mounted AVD technique have shown a reduction in anxiety when compared with the audio distraction technique and when no distraction was used. Prabhakar et al.^10^ who compared audio and AVD techniques in which VPT gave insignificant results. However, studies done by Venham et al.^13^ and Alwin et al.^8^ showed that VPT was an effective measure of the emotional state of the child at a particular instance.

During the administration of LA, a peak in anxiety was observed in the present study. This may be due to the stressful event of LA administration. Similar findings were also observed by Baldwin.^14^ Sowjanya et al.^15^ also showed that the physiological recordings were high at the time of administration of LA. The increases in the pulse rate during stressful procedures are in accordance with the studies done by Myers^16^ and Messer.^17^

Observations from the present study showed that although there was an increase in the pulse rate in all the four groups, there was a greater increase in the pulse rate during the subsequent visits in the control group and the audio group as compared to audio video groups. There was a less increase in the pulse rate in both the type of the audio–video group of distraction used in the present study indicating that both the AVD techniques were better in reducing anxiety than the audio distraction. The results were in accordance with the study done by Prabhakar et al.^10^ who also observed that there was less increase in the pulse rate in the audio–video group when compared with the audio group.

In the present study, there was no significant difference found in oxygen saturation during all the visits across the groups. Similar findings were observed by Yelderman et al.^11^

The anxiety ratings in the audio group were lower as compared with the control group but the difference was not statistically significant. Similarly, Marwah et al.^18^ and Yamini et al.^19^ found that audio distraction did a decrease in the level of anxiety in anxious pediatric dental patients although not to a significant level. However, Anderson et al.^20^ concluded from his study that patients undergoing dental restoration report less pain and discomfort when listening to music.

The results from the present study showed that AVD techniques were the most effective means of managing the anxiety in children. Similar results were found in pediatric and adult dental patients.^10,21^

Various audio and AVD techniques have been used in the literature such as audio analgesia,^22^ contingent audio tapes,^23^ music,^18,20^ television,^10^ handheld video game,^24^ videogames displayed through a virtual reality (VR) helmet,^25^ and virtual reality audio–visual eyeglasses.^26–28^

The AVD eyeglasses method is not indicated in some situations. A few children who demonstrated disruptive behavior and refused treatment immediately rejected the AVD eyeglasses. Further, AVD eyeglasses are not appropriate for children who are highly vigilant and insist on controlling the situation, also the need for maintenance and the unavailability of eyeglasses for children with small faces limit the use of AVD eyeglasses.^27^ Also, AVD eyeglasses can present as a technical obstacle that limited their access to the children's teeth. In addition, they noted that having to ensure correct positioning of the eyeglasses hampered their work.^27^

Whereas AVD by television is passive and does not hamper the patient–dentist relationship, as the operator can communicate at any point during the treatment. The child having the audiovisual presentation will have a multisensory distraction as he/she will tend to focus on the screen, and not on the dental treatment,^29^ and the sound of the program will help the child to eliminate the disagreeable dental sounds such as the sound of handpiece.^30^ Hence, two techniques using television, one being chair mounted and another being the ceiling mounted, were effective distraction techniques used in the present study. The distraction techniques were readily accepted by patients and parents. Children usually looked forward to it in the subsequent visits.

## CONCLUSION

For children of all ages and temperament, the impressions of distress left by the first dental visit, as well as the experience associated with each successive dental procedure, build memories that affect conduct on upcoming appointments. From our study, we conclude that

- Ceiling-mounted and chair-mounted AVD techniques are a novel distraction which can be an effective and alternative distraction technique for the behavior management for anxious pediatric patients.
- Distraction techniques explained in the present study are economical and widely available and can be easily installed in the departments and in private clinics to aid in behavior management.
